# Clinical impact of a multifaceted intervention aimed at decreasing distress in people living with dementia: evaluating the Reconnect program

**DOI:** 10.3389/fpsyt.2023.1191105

**Published:** 2023-12-08

**Authors:** Cheryl Baird, Hannah Miller, Kreshnik Hoti, Jeffery Hughes

**Affiliations:** ^1^Orchard Care Homes, Harrogate, United Kingdom; ^2^Faculty of Medicine, University of Prishtina, Pristina, Albania; ^3^Curtin Medical School, Curtin University, Perth, WA, Australia; ^4^PainChek Ltd., Sydney, NSW, Australia

**Keywords:** Reconnect program, Orchard Care Homes, PainChek, dementia, behavioral symptoms

## Abstract

**Background:**

To better meet the needs of people living with advanced dementia, Orchard Care Homes, United Kingdom have established an enhanced person focused program, namely the Reconnect program, which provides an enriched psycho-social care to enhance peoples’ quality of life and well-being. Here we aimed to review the impact of this program on people living with dementia.

**Methods:**

In this study the implementation of the Reconnect program was evaluated for two six-month periods (April–September 2020 and April–September 2021). The focus of this evaluation was on three key interventions: increasing meaningful occupation and engagement; improving pain identification and management, and reducing constipation. The Reconnect program was conducted in a single for-profit care home. It involved residents with complex dementia needs who previously not responded to support in alternative settings or found previous care ineffective in relieving their distress and reducing risks they pose to themselves or others.

**Results:**

A total of 24 people participated in the program during this evaluation. We observed a substantial increase in engagement in meaningful activity per person, including an increase of outdoor access to fresh air. Pain management improved as evidenced by more standardized pain assessments using the PainChek system and coverage of people with either regular and/or “when required” pain management. Constipation relief also improved. For the two comparison periods, distress responses per resident reduced from 14.5 to 10.6 events and use of regular pain relief increased from 21.7 to 48.1%. Use of “when required” benzodiazepine halved from 6 months average of 46 to 23.2 doses given. Benzodiazepine dose reductions increased from 13.3 to 31.8%, while cessations increased from 20 to 50%. We also observed a reduction from 76.3 to 56.3% in antipsychotic use. Their dose reductions increased from 8.3 to 40% and drug cessation was made in 30% of people using antipsychotics (compared to the first period in which no medication cessation was observed). A 91.7% reduction (i.e., from 36 to 3 events) in safeguarding events related to behaviors was also observed.

**Conclusion:**

Introduction of the Reconnect program, through its interventions focused on meaningful activity engagement, pain management and constipation relief resulted in substantial improvements related to people’s distress, safeguarding and psychotropic use.

## 1 Introduction

People living with dementia can have complex psychosocial unmet needs requiring specialized care. They can experience heightened levels of psychological distress which is expressed through behavioral changes. Over 90% of people living with dementia experience behavioral and psychological symptoms of dementia (BPSD) (1, 2).

Literature data reports various programs which have been introduced with the aim of providing individualized support and therefore improve the care of people living with dementia (3–8). Reconnect program is an enhanced person focused approach to caring for people with dementia with focus on maintaining a good quality of life. The program draws on a number of published models, bringing these together to create the concept of care provision. These include: (1) Tom Kitwood’s work which focuses on putting the person before the diagnosis, malignant social psychology and the importance of meeting people’s psychological needs (9), (2) The Newcastle Model (10), which forms the foundations for understanding and evaluating the causes of distress and developing individual approaches to reducing the frequency, intensity and duration of these periods of distress; and (3) The PINCH ME (**P**ain, **I**nfection, **N**utrition, **C**onstipation, **H**ydration/Dehydration, **M**edications, **E**nvironment) (11) tool for delirium which compliments the Newcastle Model. Further, the Reconnect model also has a focus on cultural views and eliminating the use of language which creates negative connotations in care staff. Finally, the environment forms part of the Reconnect program drawing on work from University of Stirling (12).

The purpose of this program is to provide specialist nursing care for people living with dementia who are experiencing persistently heightened levels of psychological distress responses expressed through their behaviors. In the Reconnect program, highly trained staff take the time to get to know each person, from their usual routines, what normal life is like for them as an individual, what brings them a sense of purpose and holds genuine meaning to them as an individual. Meaningful engagement is an integral part of the Reconnect model. Meaningful activities were periods of engagement in daily life that each person gained observed pleasure and sense of purpose from. These ranged from leisure such as arts, crafting, games; household jobs such as washing up, housekeeping, helping with laundry; outdoor garden jobs (light activities). Staff were trained to understand the importance of having deep knowledge of the person’s life which they gained from discussion with the person and or their family. They then used this to develop enhanced engagement opportunities. For example, there were “Occupation Boxes” specific to individuals and their previous job’s role, for example chemist, civil engineer or jewelry finisher. These were used to give people purpose and meaning supporting the theory that lack of purpose and meaning in their life contributes to distress (13). Through focused, individualized support, the aim is to reconnect the person to the world around them, to their identity and life. A characteristic of people participating in the Reconnect program is the fact that their previous care was ineffective in relieving their distress and reducing risks they pose to themselves or others. Staff participating in this program receive comprehensive training to evaluate complex causes of distress and develop support plans to reduce the frequency, intensity, and duration of the distress through non-pharmacological approaches. In doing so, those delivering the Reconnect program are encouraged to focus on the individual’s abilities and identify areas where the person can be supported to increase their independence and participation in daily life. As a result, the program also aims to reduce the use of psychotropic medication and safeguarding incidents (i.e., activities involving protecting people from harm and ensuring their safety and wellbeing) (14).

To achieve the above, the Reconnect program focusses on three key areas of intervention: (1) increasing meaningful occupation and engagement, (2) improving pain identification and management, and (3) reducing constipation. It was hypothesized that the above interventions may reduce the frequency, intensity and duration of people’s distress, and as a result consideration could then be given to reducing or stopping psychotropic medication and incidents reportable to safeguarding would begin to decrease.

### 1.1 Interventions introduced in the Reconnect program

It was identified from the site’s clinical practice thus far that reducing psychotropic medication use and safeguarding events could not be achieved in isolation. There were fundamental foundations which needed to be embedded to ensure reductions in psychotropic medication use were sustainable. Further, reducing safeguarding incidents would also require practice change. Therefore, to address this the Reconnect program employed three areas of focus, namely: increasing meaningful occupation and engagement; improving pain identification and management, and reducing constipation (which is more common in dementia) (15).

### 1.2 Establishment of multidisciplinary care team

A multidisciplinary care team (MDCT) was established for the purpose of providing expertise from physical and mental health and pharmacological fields, ensuring reviews of people’s current presentation was holistic and focus was not solely related to the diagnosis of dementia. It also aimed to ensure care needs assessments were equitable in line with the Equality Act 2010 and Care Quality Commission’s regulations. The Reconnect approach utilized the Newcastle Model (10) to assess causes of distress and reductions in psychotropic medication where the final element once all potential cause of distress had been evaluated and addressed, e.g., ensuring sense of purpose, choice and autonomy, pain management, holistic medication analysis for contributory factors, blood analysis and approach of caregivers.

The MDCT was comprised of external and internal staff, including members of the Older People’s Mental Health Team, a Community Psychiatric Nurse (CPN), Advanced Clinical Pharmacist, Advanced Nurse Practitioner (ANP), Dementia Lead, deputy manager and nurses. The MDCT met weekly. Nurse prescribers and general practitioners (GPs). prescribed medications, while nurses were proactive in reducing prescriptions.

The Older People’s Mental Health Team provides a specialist community mental health service to people who have mental health needs in later life. The team consist of community mental health nurses, occupational therapists, psychologists, support workers and doctors. The team offers assessment of mental health which include psychological, social, and physical components. The team assesses an individual’s needs and build an individualized care plan. Interventions may include psychosocial interventions to help with the management of symptoms associated with dementia, advice and monitoring around medication and coping strategies.

### 1.3 Increasing meaningful occupation and engagement

This involved increasing the staff to resident ratios and providing staff with training to assess people’s needs, together with modifications to the living environment as outlined below.

### 1.4 Staffing and training

Higher staffing ratios were put in place: one member of staff to three people during the day and one to five people over night plus a nurse 24 h a day. Staff, as part of their role, were trained to engage people in whatever is meaningful to them, what supports them to have a sense of purpose in their life and what helps them to reduce the frequency, intensity and duration of the distress they experience. The teams were trained on a number of significant areas, all of which can have an impact on the person living with dementia and subsequently the level of distress they experience. The 6 days training program included the following modules: Dementia Awareness, Law and Regulations, Communication, Engagement and Meaningful Occupation, The Environment, Physical Health and Dementia, Distress Responses and De-escalation, Family Support and Normalizing Life. Engagement Care Plans were introduced to document the tailor activities to be undertaken by each person, and to record their involvement in those activities.

### 1.5 The living environment

The environment was designed to offer opportunities for residents to engage as independently as possible. For example, developing a fully equipped workshop for tinkering and projects were designed as well as a laundry room with a working washing machine and whole range of associated items so people can occupy themselves with normal activities if they choose. These were put in place to function as normal life opportunities. We also aimed to increase people’s access to outdoor fresh air by 100%. This was achieved by encouraging people to contribute to the up keep of the garden.

### 1.6 Focus on pain identification and management

Considering the well-established link between pain and behavioral symptoms, including the severity of these symptoms (1) it was considered necessary that the Reconnect program put a focus on pain identification and management in order to achieve its aims. In this regard, PainChek was introduced in January 2021 with the view of addressing inconsistency in the way in which pain was identified up until then. PainChek data was then shared with the Older People’s Mental Health Team when reviewing people’s presentation and psychotropic medication.

PainChek is a multimodal, multi-platform, and hybrid pain assessment system. It is comprised of three key components namely the PainChek point of care app, PainChek analytics and PainChek training. The PainChek app uses the camera of a smart-device to capture images from a 3 s scan of a person’s face, which are analyzed in real-time using Artificial Intelligence enabled technology to detect 9 muscle movements (Action Units or AUs) which are indicative of the presence of pain. Following this analysis, the images are discarded. The facial data are then combined with non-facial pain features inputted by the user across another 5 domains [The Voice (9 features), The Movement (7 features), The Behavior (7 features), The Activity (4 features) and The Body (6 features)] via a series of digital checklists. Each feature detect is assigned a score of 1 and there are 42 features in total (16). The app uses the data from the 6 domains to automatically calculate a Pain Score and then assign a Pain Intensity based on previous calibration against the Abbey Pain Scale (17) as follows: Pain score: 0–6 = No pain, Pain Score: 7–11 = mild pain, Pain Score: 12–15 = moderate pain and Pain Score: 16–42 = severe pain.^1^ The psychometric properties of PainChek^®^ have been evaluated in people with moderate to severe dementia. Using the Abbey Pain Scale as a comparator PainChek^®^ demonstrated excellent concurrent validity (Pearson’s Correlation, *r* = 0.911, *p* < 0.01) and excellent reliability (K – 0.857; CI 0.819–0.895) (16). Once saved, this data is stored on the device until it is connected to the internet when it is synchronized to the secure cloud based PainChek analytics database.

Prior to the implementation of this pain assessment system Orchard’s staff completed education and training in the use of the PainChek system. This involved a 2 h program which includes didactic sessions which cover the following topics: (1) pain in dementia (2) pain behaviors in people with dementia, (3) pain assessment in people with dementia, and (4) the PainChek system. Further details on the PainChek system can be accessed in already published previous material (16).

### 1.7 Focus on reducing constipation

Before the commencement of the Reconnect program a review of bowel charts in early 2020 revealed that a significant number of people had constipation. Yet, it was considered that the contribution of constipation to people’s distress was minimal. As a result, staff were coached to ensure constipation is reviewed and routinely considered as a potential contributory factor to distress. It was also ensured people were not prescribed osmotic laxatives wherever possible due to their dehydrating effects. Additionally, medication with constipation side effects were reviewed regularly and deprescribed where possible. Orchard Care Homes also employed a Catering Lead in order to support adjustments in dietary fiber to improve bowel function.

In this paper we report our findings following evaluation of the above interventions with focus on their impact on distress and psychotropic medication use, as well as safeguarding events.

## 2 Methods

### 2.1 Study setting

The study was conducted in a for-profit care home comprised of 103 rooms across five communities (wings). The Reconnect community consisting of 18 rooms. The staffing ratio in the Reconnect community was 1 support worker for 3 people, plus a registered nurse on duty. In non-Reconnect communities, staffing is generally 1 carer to 6 people, 1 nurse for 20 people and in some instances a senior carer. Staffing numbers are calculated by using an in-house dependency tool based on care and support needs.

### 2.2 Participants

Residents enrolled into the Reconnect program had previously not responded to support in alternative settings or found previous care ineffective in relieving their distress and reducing risks they pose to themselves or others. They all required high levels of intensive, individualized support. They had a current presentation which included cognitive impairment causing memory function issues, disorientation to time and place, and limited insight into their risks and needs, both physical and psychological. In addition, they required trained nursing staff and a specialist care team who could prompt and provide support with daily needs including maintaining personal hygiene, eating and drinking, safe mobility and engaging positively with the world around them.

Not all residents were enrolled in the Reconnect program over the 12 months of the study. This was due to practicalities around residents moving in at different times and ongoing turnover of people coming in and leaving. Nonetheless, as soon as new residents entered the program because of their complex dementia needs their data was collated.

### 2.3 Study design

This study evaluated the performance of the Reconnect program as part of Orchard Care Homes quality improvement program. The program is overseen by the Director of Quality and Care (CB) who is responsible to ensure that the quality improvement programs bring about immediate, positive changes in the delivery of care. All data collected for the purpose of evaluating the performance of the program was data which is routinely collected as part of people’s care. All data was aggregated for the purpose of analysis, and data de-identified to ensure participant confidentiality and privacy. Based on the above, and the fact that the Reconnect program is to become a standard care model within Orchard Care Homes it was considered the evaluation did not warrant ethical review.

### 2.4 Data collection

The program was evaluated by comparing data from the 6 months April to September 2020 (i.e., the initial implementation of the Reconnect program) and April to September 2021 (i.e., following full implementation of the Reconnect program). Psychosocial interventions were initiated in May 2020. At this time staff underwent training (including remote training due to COVID-19) to deliver these interventions which was conducted over approximately 6 weeks. PainChek was introduced in January 2021 and therefore from this period onward the entire implementation of program interventions described here commenced (i.e., full implementation) Evaluation took place in one of the sites managed by this group. The 6 months data comparison was chosen to reduce the effect of nuanced monthly variations. The following were used as performance indicators of the program: (a) access to fresh air to increase by 100%, (b) reduction in use of benzodiazepines regular and “when required” (PRN) by 20%, (c) reduction of antipsychotic use by 20%, and (d) reduction in safeguarding incidents by 20%.

Periods of meaningful activities were recorded at the point of care by care staff through the electronic care system. Entries were made at the time the activity happened. That could be anytime of the day or night and the frequency of these entries was dependent on what the person chose to do. Evidence that the person had drawn psychological benefit from these was through carer/nurse observation of the person’s responses through verbal and non-verbal communication of the individual. The team were coached in understanding communication to ensure both these benefits and distress were recognized. The exposure to fresh air was documented by the care staff adding the time spend outside to the person’s electronic care record.

Distress responses were recorded on the same system, again through observations. These were not necessarily safeguarding incidents. Safeguarding data relates to incidents which were referred to the local Safeguarding Adult Board (SAB) (18). Reports were sent to the SAB for review to ensure transparency. Not all reported incidents resulted in the SAB opening a safeguarding review.

The electronic care plan system allows staff to use the Bristol Stool Chart (19) to record frequency and consistency of bowel motions. This is then presented in a timeline which is used to determine if the individual is potentially constipated.

The method of collate the figures involved a monthly review of data on the electronic care plan system. This was a count of events, where the qualitative entries on the care planning system were translated into quantitative data by simply counting number of relevant entries. These data were then used together with the numbers of periods of engagement, incidents of distress, safeguarding reports, minutes of fresh air, prescriptions of pain relief, constipation relief, antipsychotics, benzodiazepines, the frequency of use of these medications and reductions/increases in doses and use. Reliability of figures was based on the team entering the information in the home. Orchard’s Head of Dementia (HM) was responsible for collating the data reducing risk of methodology variation.

### 2.5 Data analysis

Data was collected on a monthly basis and included information on people’s age and gender, dementia diagnosis, potential cause of distress, medication prescriptions and safeguarding incidences. Descriptive statistics were analyzed using Microsoft Excel™ *Vs* 16.70.

## 3 Results

### 3.1 Demographics of participants

Starting with 16 people, a total of 24 people participated in the Reconnect program during the evaluation period, with a maximum of 18 people within the program at any one time. These 24 people were made up of 14 males and 10 females, and their average age (standard deviation) was 81.2 (6.3) years, ranging from 67 to 92 years. People were living with a range of dementia types including Alzheimer’s Disease (*n* = 8), Vascular Dementia (*n* = 9), Unspecified Dementia (*n* = 3), Mixed Dementia (*n* = 1), Lewy’s Body Dementia (*n* = 1), Frontotemporal Dementia (*n* = 1), and Charles Bonnet Syndrome (*n* = 1).

### 3.2 Multidisciplinary team reviews

It is worth noting that January 2021 saw the first holistic MDT meeting. The establishment of the team resulted in every person having an antipsychotic review regularly, with the maximum interval of review in the study being 6 weeks in line with NICE guidance (20).

### 3.3 Meaningful occupation and engagement

**Figure 1:**
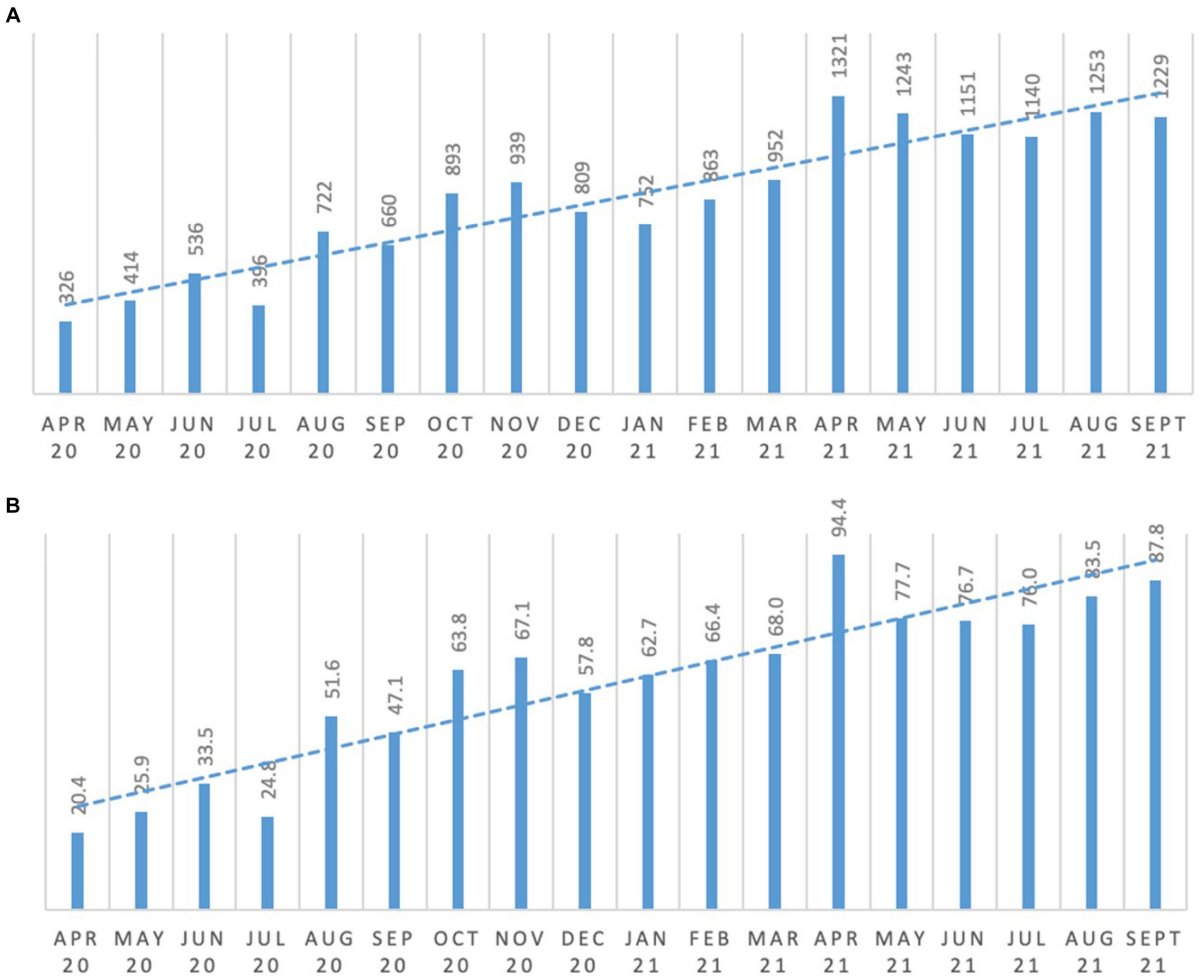
Meaningful occupation and engagement. **(A)** total number of engagement/activity; **(B)** average engagement frequency per resident.

The introduction of the Reconnect program saw people increasingly engaging in meaningful activity and occupation consistently over the period of the study as shown in Figure 1. Comparing the six-month average from April to September 2020 and April to September 2021, engagement in meaningful activity per person increased by 144%.

### 3.4 Access to fresh air

**Figure 2:**
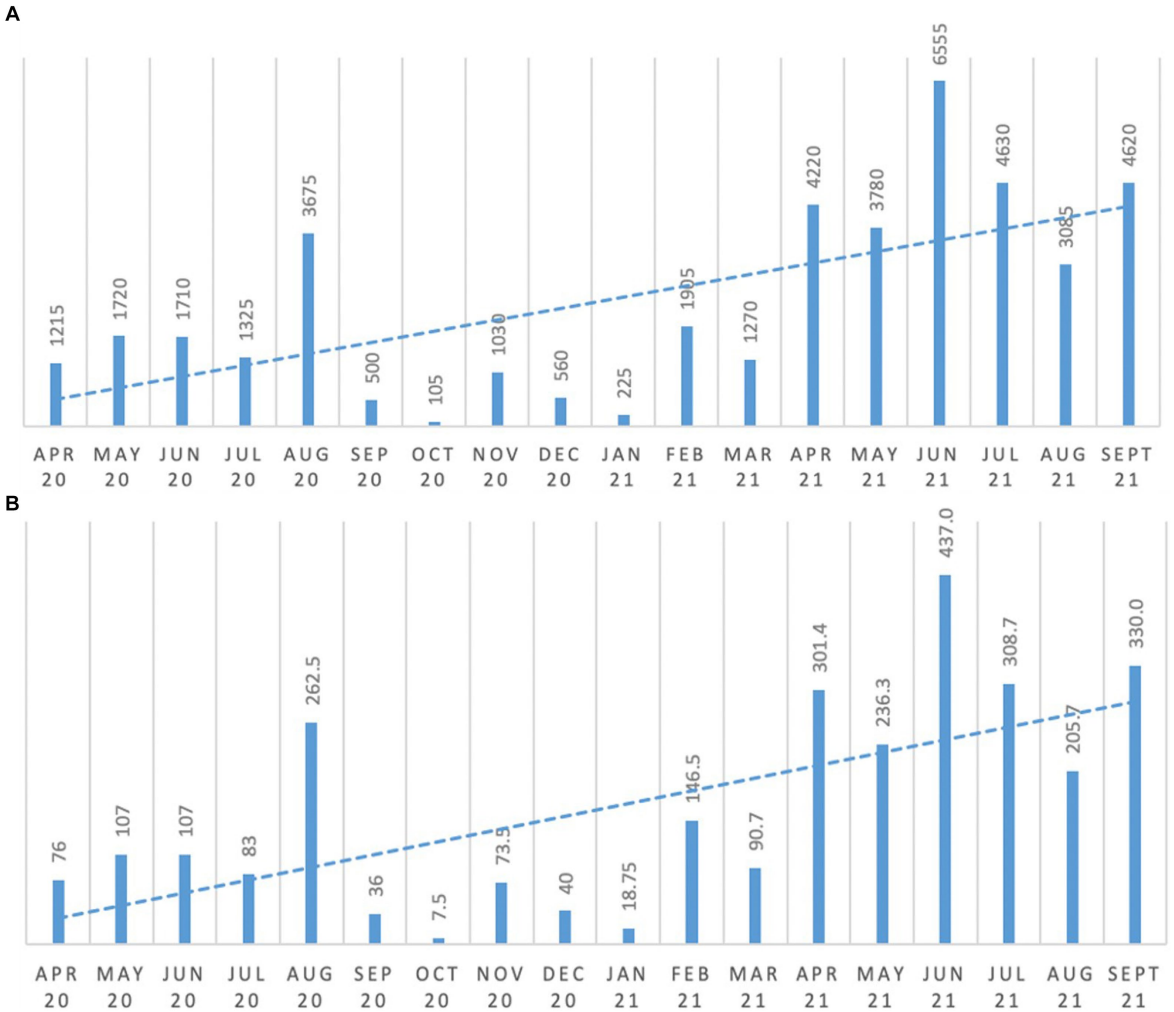
Changes in residents’ exposure to fresh air. **(A)** minutes of fresh air for all persons; **(B)** average minutes of fresh air per person.

As can be seen from Figure 2 there was (a) an increase in the total number of minutes all people accessed outdoor fresh air spaces, as well as (b) an increase in the average minutes of fresh air access per person. Comparing the six-month average from April to September 2020 and April to September 2021, access to fresh air per person increased and exceeded the 100% increase target. For example, as can be seen in Figure 2B, average minutes of fresh air per person increased from 111.9 min (April 2020 to September 2020 average per person), to 303.2 min (April 2021 to September 2021 average per person).

Lower figures can be seen during colder months and a period when work was being done in the garden, which restricted access.

### 3.5 Pain management

**Figure 3:**
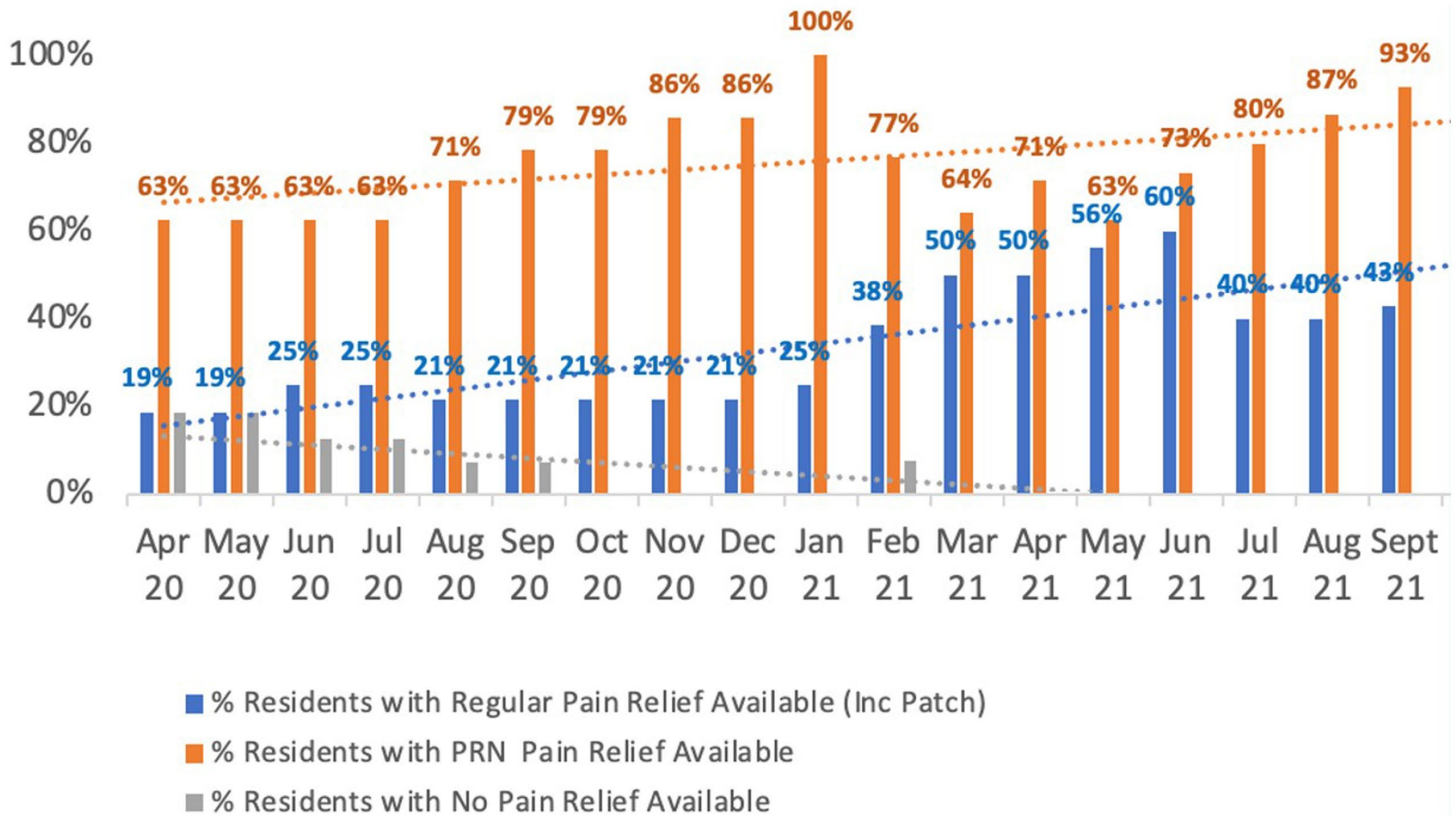
Overall availability of pain relief.

In April 2020, at the commencement of the Reconnect Program, 19% of people had no available pain relief. Further, pain identification was not approached in a consistent manner (i.e., there was no standardized tool being used for assessing pain in people living with dementia). Since the introduction of the PainChek system, assessment results were regularly shared with members of the Older Adults Mental Health Team when reviewing people’s presentation and psychotropic medications. This resulted in vast majority of people, as of September 2021 having regular medication, PRN medication or a combination of both for pain management (Figure 3). As can be seen in Figure 3, more people were put on regular pain relief after January 2021, which corresponds with the introduction of the PainChek system in the Reconnect program. In this regard, when comparing the first 6 months (April to September 2020) of the program (i.e., initial implementation) to the same 6 months 12 months later (April to September 2021) when full implementation had occurred, it can be seen that the regular use of pain relief treatment increased from a 6 months average of 21.7% (initial implementation) to a 6 months average of 48.1% (full implementation).

### 3.6 Constipation relief

**Figure 4:**
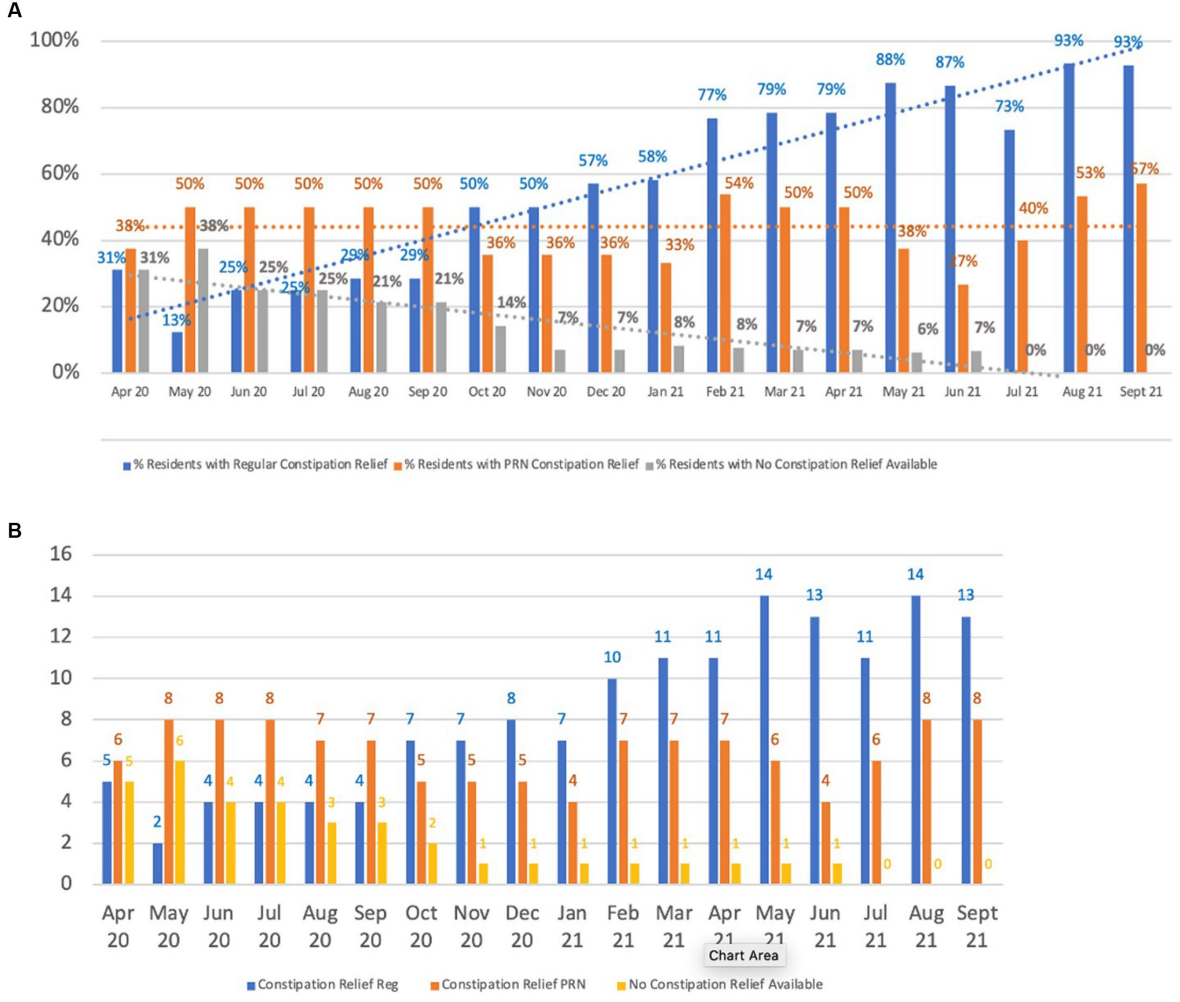
Constipation relief prescriptions. **(A)** Availability of constipation relief, **(B)** Constipation relief prescriptions.

As can be seen from Figure 4, focusing on reducing constipation resulted in a significant shift in treatment of constipation, with almost all people as of September 2021 having a prescribed laxative available. For example, in the first comparison period (April 2020 to September 2020), cumulatively a total of 26.8% of residents had no constipation relief available compared to only 3.3% in the period between April 21 to September 2021 reaching no resident without constipation relief in the second half of this period (Figure 4A). Decisions to prescribe laxatives were guided by a review of Bristol Stool Chart data by the clinician and multidisciplinary team. The focus was to evaluate whether constipation was a potential contributor to the person’s distress.

### 3.7 Distress responses

**Figure 5:**
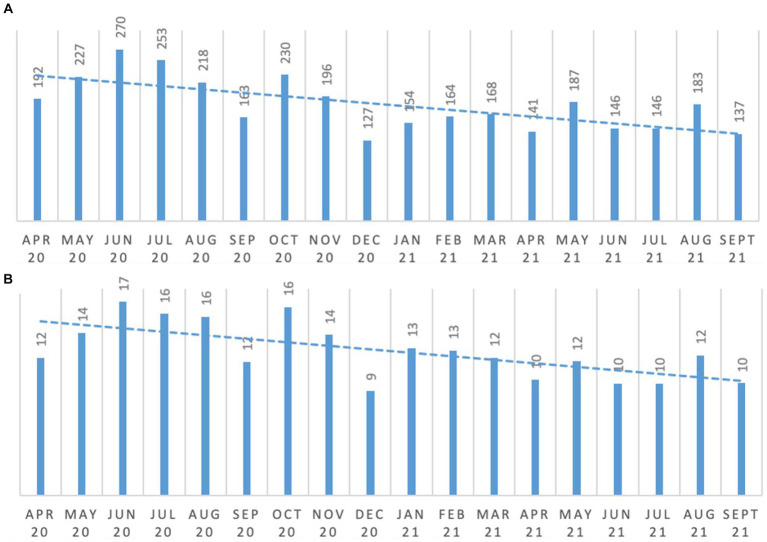
Occurrence of distress responses. **(A)** Overall distress responses; **(B)** average distress response per resident.

Initially the expectation was that reporting of distress responses would increase as the care team were coached on effective recording. However, as shown in Figure 5 this did not occur, in fact over time there was a decrease in total distress responses. This has accompanied an improvement in the quality of reports with detail being noted on what may be causing the distress and interventions being used to reduce distress when it occurred.

Fluctuations seen in the incidents of distress are inevitable as new people move into the care home. As can be seen in Figure 5B, by September 2021 average distress responses per person were reducing (i.e., from 14.5 to 10.6 events for the period April 2020 to September 2020 and April 2021 to September 2021, respectively). Further, the vast majority of distress response reports indicated minimal distress suggesting the effective use of psycho-social approaches may reduce distress event frequency, intensity and duration.

### 3.8 Psychotropic medication use

#### 3.8.1 Benzodiazepines

**Figure 6:**
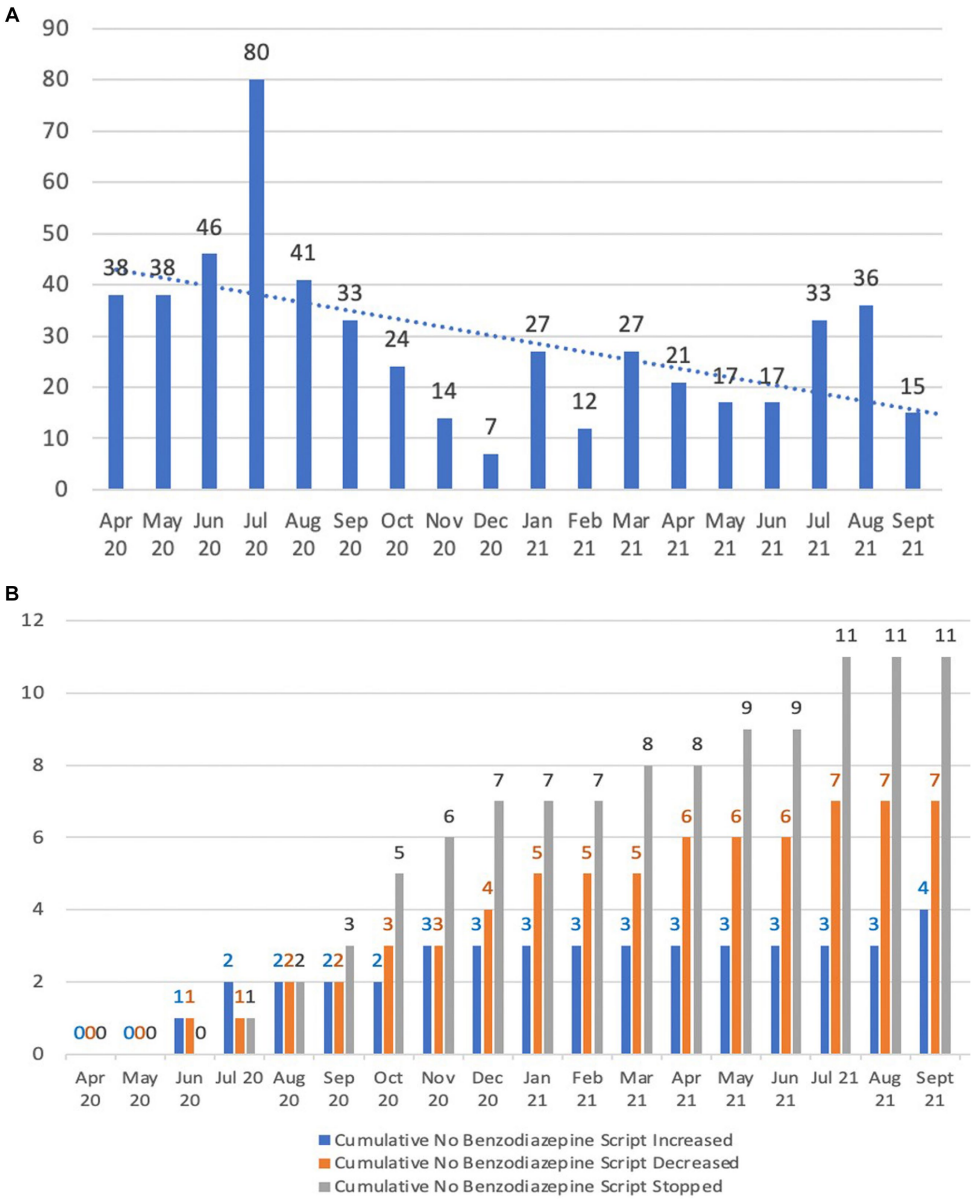
Benzodiazepine usage patterns. **(A)** Benzodiazepine PRN doses given; **(B)** Cumulative benzodiazepine scripts changed.

When comparing the average use of benzodiazepine in the six-month period April to September 2020 to the same six-month period in 2021, total use of PRN benzodiazepines halved from 46 to 23.2 doses given (Figure 6A). Benzodiazepine dose reductions increased from 13.3 to 31.8% (i.e., 2 out of 15 residents who were using benzodiasepines had their doses reduced by the end of September 2020 in comparison to 7 out of 22 residents by the end of September 2021), while the percentage of people who had them ceased increased from 20 to 50% (i.e., from 3 out of 15 residents by the end of September 2020 to 11 out of 22 residents by the end of September 2021). This is illustrated in Figure 6B.

#### 3.8.2 Antipsychotics

**Figure 7:**
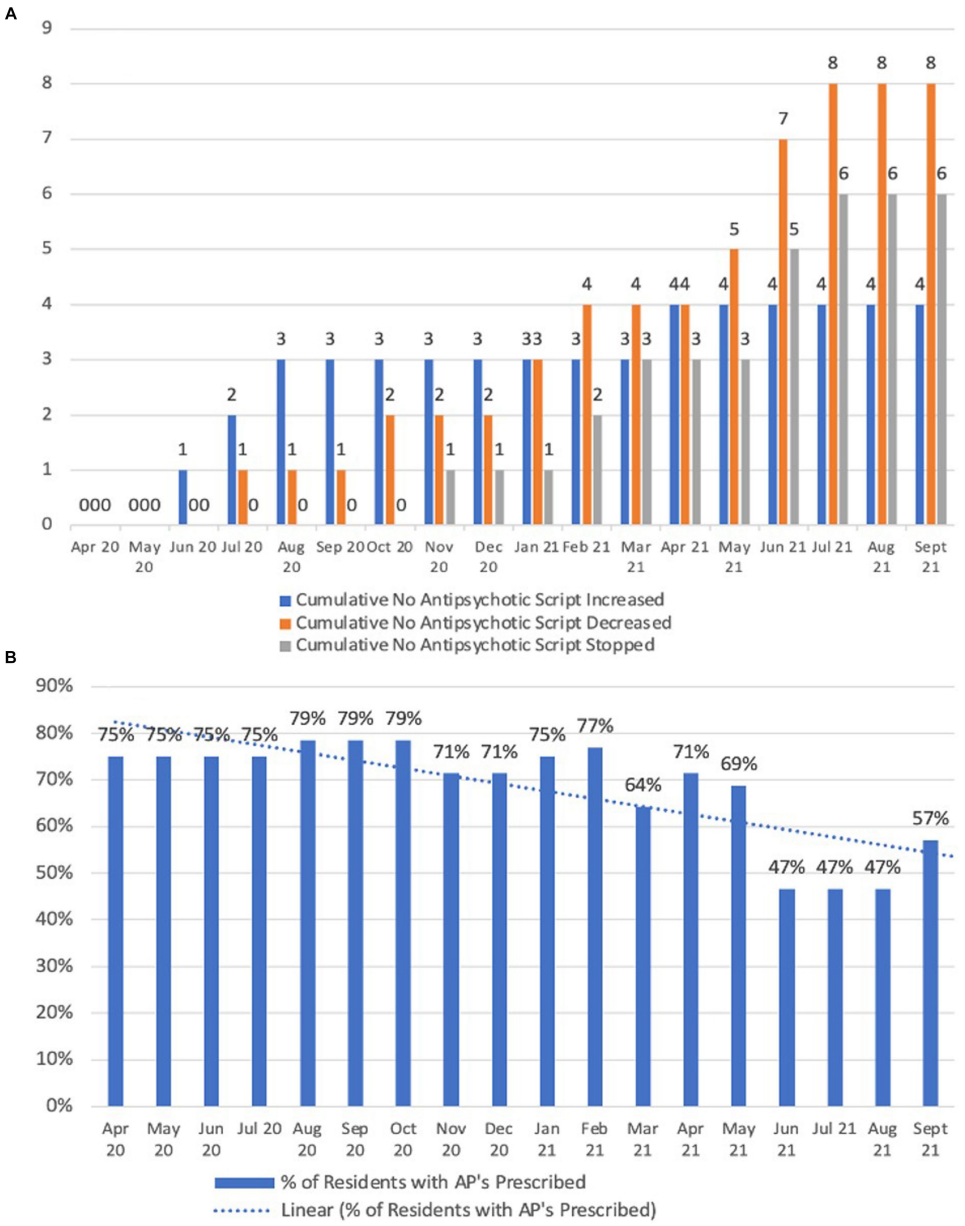
Antipsychotic usage patterns. **(A)** Cumulative antipsychotic scripts changed; **(B)** Percentage of residents with prescribed antipsychotic medications.

Following the commencement of the Reconnect program, there was a reduction in the prescribing of antipsychotic agents as shown in Figure 7. Antipsychotic dose reductions during the April 2020 to September 2021 period in comparison to the same period during 2021 increased from 8.3 to 40% (i.e., 1 out of 12 residents using antipsychotics had their dose reduced by the end of September 2020 in comparison to 8 out of 20 residents by the end of September 2021), as shown in Figure 7A. There were no cases of antipsychotic cessation during April 2020 to September 2020, in comparison to the same period during 2021 in which these medications were ceased in 30% of residents taking them (i.e., 0 out of 12 people had their antipsychotic ceased by the end of September 2020 compared to 6 out of 20 people by the end of September 2021). Overall, percentage of residents who were prescribed an antipsychotic was reduced from a 6 months average of 76.3% to a 6 months average of 56.3% in the period April 2021 to September 2021 (Figure 7B).

### 3.9 Safeguarding

**Figure 8:**
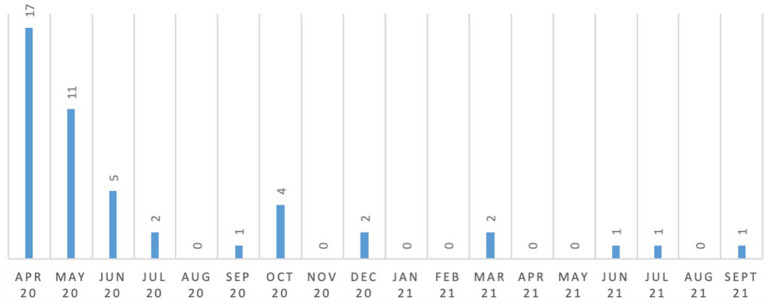
Safeguarding events reported (behavior related).

Comparing the 6 month periods from April to September 2020 and April to September 2021, there was a 91.7% reduction in the quantity of safeguarding events reported which were behavior related (i.e., a total of 36 safeguarding events were reported during six-month period from April 2020 to September 2020 in comparison to only 3 events in the same period during 2021). More details have been provided in Figure 8.

## 4 Discussion

In this study we report our findings from evaluating two six-month periods of the Orchard Care Homes’ Reconnect program. Our findings suggest that the introduction of this program over time led to important improvements in people’s distress, safeguarding events, psychotropic use and life experience. The Reconnect program started in April 2020, which is the time when changes were started to be measured. The implementation of the program continued and the performance indicators for program started to show gradual improvement over time as the program’s implementation matured. The figures provided in our findings illustrate these positive and gradual improvements. There are a number of reasons leading to these improvements. The Reconnect program switches the focus back to the individual and their needs. This enhanced person focused approach is based on the premise that activities or interventions that are tailored to address an individual’s needs are more likely to engage the person with dementia and therefore be more enjoyable and meaningful for them (21). This approach removes them from a care process that dictates what they will do, and which assumes it meets their needs and is engaging, to one that is tailored for the individual. It employs meaningful occupation which has been defined as: “Any task or activity that is important and meaningful to the person with dementia” (22). The results of this study demonstrated an increase (i.e., 144%) in people’s engagement in meaningful activities. It should be emphasized that to achieve this the care team were coached in developing engagement approaches that were individualized for each person. Key in facilitating the observed increase in engagement in meaningful activity was the introduction of Engagement Care Plans which were non-existent prior to the introduction of the program. Additionally, it is worth mentioning that increasing access to outdoor fresh air activities, which was one of the key strategies employed in this program also increased significantly and beyond target. It has been well evidenced by other studies that getting people with dementia “back to nature,” in this case participating in outdoor activities, has positive effects in their overall care, including people’s mood, social interaction and other behaviors (23, 24).

It has been postulated that distress response behaviors, may arise from people with dementia having unmet needs (e.g., meaningful activity and social interaction) that they are otherwise unable to express (25). Failure to address the unmet need(s) of a person living with dementia provides an ongoing source of distress which can manifest in behavioral change. There are many other causes of distress amongst people living with dementia including environmental factors, physical and sensory impediments, issue of nutrition and hydration, psycho-social factors, and medical conditions (e.g., constipation, pain, and urinary tract infection) (26). As such, it is important these are also considered and addressed, as they may negatively impact on an individual’s ability to participate in activities that they enjoy. As pain and constipation are common causes of distress response behaviors amongst people with dementia, it was decided that these should be addressed as part of the Reconnect program also. It is well known that failure to recognize and treat pain effectively is a well-known problem amongst people living with dementia (16, 27). Pain is a trigger of distress, yet often overlooked as a contributing factor, and the person is not only left to suffer unnecessarily in pain, but also to be prescribed psychotropic agents to manage their behaviors (28). It has been demonstrated that systematic use of observational pain assessment tools can improve the identification of pain in people with dementia, positively impacting the use of non-pharmacological pain interventions and analgesics (1, 29, 30). Savvas et al. evaluated an evidence-based program involving staff education and training in the use of three observational pain assessment tools (i.e., Abbey Pain Scale, Pain Assessment in Advanced Dementia [PAINAD], Non-communicative Patient’s Pain Assessment Instrument [NCPI]) and revised pain management procedures. As a result of the program analgesic use improved, with fewer people receiving no analgesics (from 15 to 6%) and more receiving regular plus as-needed analgesics (from 24 to 43%). These changes in analgesic use were associated with improvements in pain relief (31).

In our study, implementation of PainChek pain assessment system resulted in standardization of pain assessments, a practice widely supported in the literature (32, 33). As was the case with Savvas et al., the introduction of PainChek was associated with an increased percentage of people receiving pain assessment as part of standard practice, and an increase in people prescribed regular and PRN analgesics. Further there was an increased scrutiny of pain assessment results by the Older People’s Mental Health Team when reviewing people’s distress and the need for the use of psychotropic agents.

Tampi et al. (30), in their review reported that that the use of analgesics appeared be well tolerated and their use was associated with reduction of BPSD. Improvements were observed in social as well as verbal and physical behaviors. These benefits were attributed to analgesic effects of medications which reduces distress and discomfort associated with pain sensation. Improvements in behavioral symptoms following pain management were also reported by other review studies as well (34, 35). Considering the above, it can be deduced that introduction of the PainChek system contributed towards improved pain assessment and management and hence, as part of this multifaceted intervention in the Reconnect program, also contributed towards reduction of distress in people living with dementia. Additionally, our findings support the clinical utility of using the PainChek system within a multifaceted program such as the Reconnect.

Constipation was also targeted as a modifiable cause of distress, as part of the Reconnect program. It is a common condition amongst the elderly and may significantly negatively impact quality of life. Various reports suggest that constipation is closely related to dementia (36–38). Recently, Wang et al. (15) reported that in individuals 65 years and older the prevalence of chronic constipation was 19.2% in those with dementia compared to 14.8% for all those aged 65 years or older. It has, however a higher prevalence amongst the elderly admitted to long-term care facilities (up to 80%) (39, 40).

People with advanced dementia may have difficulty verbally expressing pain and discomfort resulting from constipation. If overlooked, constipation may lead to behavior and psychiatric symptoms of dementia (BPSD) including aggressive behavior (41). Such behavior leads to increased carer burden and significant physical and psychological stress (1, 42). In light of this, staff in the Reconnect program were trained to consider constipation as a possible cause of people’s distress, and as a result there was a focus on improving people’s bowel care. Additionally, it should be noted that while we have seen a shift in constipation treatment and laxative availability, the ultimate aim was to avoid constipation and therefore reduce the need for use of ongoing laxatives. Supporting the focus of the Reconnect program on bowel care are the results of a recent study by Naito et al. (43) which demonstrated this can result in multiple benefits including improved quality of life, improvements in people’s distress related behaviors and reduced caregiver distress.

Our study has a number of limitations, including the fact that the Reconnect program was evaluated on one site only and there was a limited number of people living with dementia who participated in the study. Additionally, it should be noted that while staff did their best to observe and therefore record individuals’ activities methodically, there is the possibility that some of a person’s activities, including events related to issues such as distress, may not have been captured due to various reasons including potential staff distractions at work or the need to attend to others. Additionally, potential confounding effects such as other medications and medical conditions which may have affected our findings and therefore people’s experience with the program. Finally, the study did not compare data against a 6 month period of time during which none of the interventions of the Reconnect program were in place, thus allowing a clearer evaluation of the effects of the interventions. Nonetheless, our findings report data from April 2020 during which time the psychosocial interventions were as yet not rolled. From that baseline, we observed gradual positive improvements in people’s pain management, distress and constipation, as well as in use of analgesic and psychotropic medications. Associated changes in people’ behaviors allowed a reduction in the use of psychotropic medications and safeguarding events. As this was a multifaceted intervention, it is not possible to assign a quantum of change to any single component, rather the results suggest that collectively these interventions provide meaningful clinical benefits and are compatible to be used concurrently within a dementia care program such as the Reconnect. We acknowledge however, that a controlled clinical trial would be required to provide definitive proof of the benefits of the program.

Notwithstanding, the aforementioned limitations, the program continues and four more Reconnect communities have been opened. Placements within these communities are generally funded by the NHS, however people may pay privately to live in a Reconnect community, if they meet the admission criteria. As the prevalence of dementia increases it is likely that more specialist services like Reconnect will be required, therefore commissioned and funded through the NHS.

Costs involved providing the Reconnect program included the organization remodeling existing sections of a care home environment prior to the service opening; training of care staff by an internal dementia lead and increased staff ratios.

## 5 Conclusion

This study provides support for the benefits Orchard Care Home’s Reconnect program targeted at reducing distress in people with dementia, through encouraging meaningful activity and engagement, and managing pain and constipation effectively. Our findings also highlight the clinical utility of PainChek as part of the targeted multifaceted program, with positive impacts on pain identification and management practices. This when combined with activities to improve meaningful occupation and engagement, and reducing constipation was associated with significant reductions in distress and subsequent psychotropic medication use and safeguarding events. While encouraging, these findings require confirmation through controlled clinical trials.

## Data availability statement

The raw data supporting the conclusions of this article will be made available by the authors, without undue reservation.

## Author contributions

CB, KH, and JH conceived the study. CB and HM collected the data. JH, KH, and CB analyzed and interpreted the data. KH and JH wrote the first draft of the manuscript. CB, HM, KH, and JH critically reviewed the manuscript. All authors contributed to the article and approved the submitted version.

## Footnotes

^1^ www.painchek.com
